# Efficacy of early enteral nutrition support on the nutritional status of patients after gallstone surgery

**DOI:** 10.1371/journal.pone.0314659

**Published:** 2025-02-12

**Authors:** Fang Wang, Yun Gao

**Affiliations:** Department of Hepatobiliary Surgery, Wuhan No.1 Hospital (Wuhan Hospital of Traditional Chinese and Western Medicine), Wuhan, China; Zanjan University of Medical Sciences, ISLAMIC REPUBLIC OF IRAN

## Abstract

**Objective:**

To evaluate the overall efficacy of early enteral nutrition support on the nutritional status of patients following gallstone surgery, providing a theoretical basis for its application in postoperative care.

**Methods:**

A retrospective analysis was conducted on 98 hospitalized patients who underwent gallstone surgery between February 2021 and March 2023. The patients were divided into two groups: the study group, which received early nutritional intervention, and the control group, which started nutritional support three days post-surgery. The primary objective was to assess nutritional status, while secondary objectives included gastrointestinal function, immune function markers, and the incidence of postoperative and gastrointestinal complications. Nutritional status, gastrointestinal function, immune function, and complications were compared between the two groups before and one week after intervention.

**Results:**

After the intervention, the study group showed significantly higher levels of total protein (TP), prealbumin (PAB), albumin (ALB), transferrin (TF), and body mass index (BMI) compared to the control group (P<0.05). Additionally, the scores for Nutrition Risk Screening 2002 (NRS 2002) and the Malnutrition Universal Screening Tool (MUST) were notably lower in the study group (P<0.05). Postoperatively, the study group experienced shorter times to first exhaust, defecation, bowel sound recovery, first meal, and overall hospital stay compared to the control group (P<0.05). Although the pre-intervention levels of CD3+, CD4+, CD8+, and CD4+/CD8+ were comparable between the groups (P>0.05), these immune markers were significantly higher in the study group post-intervention (P<0.05). The incidence of both postoperative and gastrointestinal complications was significantly lower in the study group than in the control group (both P<0.05).

**Conclusion:**

Early enteral nutrition support significantly improves the nutritional status of patients after gallstone surgery, enhances gastrointestinal and immune function, and reduces the occurrence of complications. These findings underscore its clinical significance and value in postoperative care.

## Introduction

Gallbladder stones are a common form of gallbladder disease in clinical practice, with gallbladder colic as the primary symptom, significantly impacting patients’ daily lives [1]. The standard clinical treatment for cholecystolithiasis usually involves surgical procedures, such as cholecystectomy or laparoscopic surgery [2]. However, the stress induced by general anesthesia and surgical manipulation during these procedures can impair the gastrointestinal blood supply, leaving the patient’s body in a state of stress. In such cases, parenteral nutrition, which bypasses the gastrointestinal tract, is less conducive to the stabilization of postoperative gastrointestinal function and may negatively affect the patient’s nutritional status [3]. Therefore, it is crucial to explore effective and safe interventions to restore gastrointestinal function and improve patients’ nutritional status as quickly as possible.

Enteral nutrition is a commonly used method of nutritional support in clinical settings [4]. According to the guidelines of the European Society of Clinical Nutrition and Metabolism (ESPEN), enteral nutrition is recommended as the first choice for nutritional treatment, while parenteral nutrition should only be considered when enteral nutrition is not feasible [5]. Studies have shown that early severe patients are prone to stress-induced hypercatabolism, which can lead to severe malnutrition [6]. Prolonged parenteral nutrition can also result in intestinal mucosal atrophy, infection, and damage to the intestinal lining [7, 8]. In contrast, early enteral nutrition support has been shown to effectively inhibit hypercatabolism, improve patients’ nutritional status, and prevent stress ulcers [5]. Additionally, enteral nutrition supports the maintenance and improvement of intestinal mucosal barrier function, reduces abnormal intestinal permeability, and prevents intestinal mucosal atrophy [9, 10].

Currently, there is limited clinical research on the use of early enteral nutrition support for patients following gallstone surgery, and existing studies present conflicting conclusions. Ge’s study demonstrated that enteral nutrition support was more effective in treating digestive tract diseases [11]. Conversely, Lin suggested that parenteral nutrition support intervention holds high clinical value in postoperative general surgery patients, citing its simplicity, safety, reliability, and ability to expedite intestinal function recovery and reduce complications [12].

To address this gap in knowledge, the present study aims to investigate and evaluate the efficacy of early enteral nutrition support on the nutritional status of patients after gallstone surgery.

## Materials and methods

### General information

A retrospective analysis was conducted on 98 patients hospitalized after gallstone surgery from February 2021 to March 2023. Data were accessed for research purposes in July 2023. The participants were divided into two groups: the study group (n = 49) and the control group (n = 49). The sample size calculation formula used was: N = (μ1−α/2 + μ1−β)^2^ * s^2^ (1 + 1/k) / (μt—μc)^2^ = 50. One patient did not meet the inclusion criteria and was excluded, leaving 49 patients in each group. This study was approved by the Institutional Review Board of the Ethics Committee of Wuhan No.1 Hospital and was conducted in full compliance with the Helsinki Declaration of the World Medical Association.

### Selection criteria

*Inclusion Criteria*:

1. Patients diagnosed with gallbladder stones based on imaging (B-ultrasound and CT) and meeting relevant surgical indications [13].
2. Patients who underwent laparoscopic surgery.
3. Patients with normal cognitive function and communication skills.
4. Patients with a Nutrition Risk Screening 2002 (NRS2002) score ≥ 3 points.
5. Patients with common bile duct stones less than 1.5 cm in diameter.

*Exclusion Criteria*:

1. Patients with systemic diseases, including hypertension and diabetes.
2. Patients who withdrew from the study midway.
3. Patients with abnormal liver or kidney function.
4. Patients diagnosed with severe mental illness.
5. Patients with contraindications to enteral nutrition or related drug allergies.
6. Patients with hemorrhagic or immune system diseases.
7. Patients with heart, brain, or kidney dysfunction.
8. Patients who had previously undergone biliary surgery.

### Methods

#### Laparoscopic surgery

During surgery, patients were positioned on their back with their head elevated and body tilted 20 to 30 degrees to the left. Several incisions (1-5mm) were made at the right midline of the clavicle, the right armpit, under the xiphoid process, and under the umbilicus. Laparoscopic exploration was performed to separate the gallbladder and arteries, using titanium clips to cut off the gallbladder duct and artery. The removed gallbladder tissue was placed in a bag and extracted through an abdominal puncture, followed by drainage tube insertion and wound suturing.

The study group received early enteral nutrition support. Within 24 hours post-surgery, 250mL of physiological saline was administered into the gastrointestinal tract at a rate of 20mL/h. On the second day, 250mL of warm boiled water and 500mL of enteral nutrition suspension (Nutricia Pharmaceutical Co., Ltd.) were administered through a nasointestinal tube, with 40-60mL given every hour. On the third day, 500mL of warm water and 1000mL of enteral nutrition were delivered in the same manner. From the 6th day, patients transitioned to semi-liquid diets, gradually increasing food intake with the recovery of gastrointestinal function.

The control group received 1440mL of fatty milk amino acid glucose injection daily via the central vein for the first three postoperative days. Enteral nutrition was introduced from day 3 to day 6, following the same schedule as the study group after the 6th day.

### Observation indicators

The main objective was to assess nutritional status, while secondary objectives included gastrointestinal function, immune function, and the incidence of postoperative and gastrointestinal complications.

#### Nutritional status

Before and one week after intervention, 4mL of fasting venous blood was collected and centrifuged to measure serum levels of total protein (TP), prealbumin (PAB), albumin (ALB), and transferrin (TF) using enzyme-linked immunosorbent assay. Nutritional status was also assessed using the NRS2002 scale [14] and the Malnutrition Universal Screening Tool (MUST) [15], which measure disease severity, nutrition, and age scores, along with BMI and weight loss.

#### Gastrointestinal function

The time to first postoperative exhaust and defecation, bowel sound recovery, first meal, and total hospital stay were recorded for both groups.

#### Immune function

Fasting venous blood samples were collected before and one week after intervention to assess CD3+, CD4+, CD8+, and CD4+/CD8+ levels using flow cytometry.

#### Postoperative and gastrointestinal complications

The incidence of complications, including infections, electrolyte imbalances, nausea, vomiting, bloating, diarrhea, and gastric retention, was recorded.

### Statistical analysis

Data were processed using SPSS 21.0 statistical software. Normally distributed data were presented as mean ± standard deviation (± s) and compared using independent sample t-tests or one-way ANOVA. Non-normally distributed data were presented as medians (quartiles) [M (P25, P75)] and analyzed using Mann-Whitney or Kruskal-Wallis tests. Categorical data were presented as case numbers (percentages) and compared using chi-square or Fisher’s exact tests. Statistical significance was set at P<0.05.

## Results

### Comparison of general information

In the study group, there were 27 male and 22 female participants, with ages ranging from 31 to 68 years and a mean age of 49.65 ± 11.28 years. Similarly, the control group included 28 male and 21 female participants, aged between 30 and 66 years, with a mean age of 49.59 ± 11.83 years. No significant differences were observed between the two groups regarding gender, age, smoking habits, comorbidities, number of stones, gallbladder wall thickness, intraoperative blood loss, operative duration, or operative success rate (P > 0.05), as detailed in Table 1.

**Table 1:** Comparison of general information between early enteral nutrition group and control group.

Parameter	Study group (n = 49)	Control group (n = 49)	t/χ^2^	*P*
Male (n)	27 (55.10%)	28 (57.14%)	0.041	0.839
Age (year)	49.65±11.28	49.59±11.83	0.026	0.979
Smoking	8 (16.33%)	6 (12.24%)	0.333	0.564
Comorbidity				
Diabetes	11 (22.45%)	9 (18.37%)	0.251	0.616
Hypertension	9 (18.37%)	8 (16.33%)	0.071	0.479
Number of stone (n)			0.234	0.628
Single	37 (75.51%)	39 (79.59%)		
Multiple	12 (24.49%)	10 (20.41%)		
Gallbladder wall thickness (mm)	6.68±1.65	6.59±1.76	0.261	0.795
Intraoperative blood loss (ml)	26.83±2.31	26.32±2.48	-1.053	0.295
Operative duration (min)	172.29±34.23	168.07±35.76	-0.597	0.552
Operative success rate	49 (100%)	49 (100%)	-	-

Note

*P < 0.05 indicates statistically significant difference. χ^2^ is the statistic value of the Chi-square test, and t is the statistic value of the T-test.

### Comparison of nutritional status

Prior to the intervention, there were no significant differences in the levels of total protein (TP), prealbumin (PAB), albumin (ALB), transferrin (TF), NRS 2002 score, MUST score, and BMI between the two groups (P > 0.05). After the intervention, the levels of TP, PAB, ALB, TF, and BMI in the study group were significantly higher than those in the control group, while the NRS 2002 and MUST scores were significantly lower in the study group (P < 0.05) (see Table 2).

**Table 2:** Comparison of nutritional status between early enteral nutrition group and control group.

Parameter	Time	Study group (n = 49)	Control group (n = 49)	t/χ^2^	*P*
TP (g/L)	Before intervention	72.98±8.87	73.07±9.08	-0.051	0.961
	One week after intervention	71.32±9.07	66.98±9.12	2.362	0.021*
PAB (mg/L)	Before intervention	162.59±12.18	163.07±11.97	-0.221	0.826
	One week after intervention	139.76±13.26	103.29±14.02	13.229	<0.001*
ALB (g/L)	Before intervention	42.89±7.89	43.07±8.65	-0.108	0.914
	One week after intervention	41.96±6.76	38.06±6.92	2.822	0.006*
TF (g/L)	Before intervention	1.67±0.23	1.64±0.29	0.567	0.572
	One week after intervention	2.76±0.31	1.92±0.34	12.781	<0.001*
NRS 2002 score	Before intervention	4.12±0.56	4.19±0.69	-0.551	0.583
	One week after intervention	2.07±0.67	2.91±0.73	5.934	<0.001*
MUST score	Before intervention	0.62±0.09	0.60±0.07	1.228	0.222
	One week after intervention	0.61±0.06	0.71±0.09	6.472	<0.001*
BMI (kg/m^2^)	Before intervention	20.01±2.18	20.12±2.43	0.236	0.814
	One week after intervention	22.86±1.97	22.01±2.09	-2.072	0.041*

Note

*P < 0.05 indicates statistically significant difference. χ^2^ is the statistic value of the Chi-square test, and t is the statistic value of the T-test. Abbreviation: serum total protein (TP), prealbumin (PAB), albumin (ALB), and transferrin (TF), Nutrition Risk Screening 2002 (NRS 2002), Malnutrition Universal Screening Tool (MUST), Body Mass Index (BMI).

### Comparison of gastrointestinal conditions

Following the intervention, the time to the first postoperative exhaust and defecation, bowel sound recovery, first meal, and hospital stay were significantly shorter in the study group compared to the control group (P < 0.05), as shown in Table 3.

**Table 3:** Comparison of nutritional status between early enteral nutrition group and control group.

Parameter	Study group (n = 49)	Control group (n = 49)	t/χ^2^	*P*
Time to first exhaust (h)	39.87±4.01	48.76±4.09	-10.864	<0.001*
Time to bowel sound recovery (h)	29.76±3.28	36.18±3.26	-9.718	<0.001*
Time to first defecation (h)	48.78±5.32	55.67±5.51	-6.297	<0.001*
Hospital stays (d)	6.67±1.51	8.47±1.47	-5.979	<0.001*

Note: *P < 0.05 indicates statistically significant difference. χ^2^ is the statistic value of the Chi-square test, and t is the statistic value of the T-test.

### Comparison of immune functions

While the levels of CD3+, CD4+, CD8+, and CD4+/CD8+ were comparable between the two groups before the intervention (P > 0.05), these levels were significantly higher in the study group after the intervention (P < 0.05) (refer to Table 4).

**Table 4:** Comparison of immune function between early enteral nutrition group and control group.

Parameter	Time	Study group (n = 49)	Control group (n = 49)	t/χ^2^	*P*
CD3^+^ (%)	Before intervention	52.98±6.02	52.65±6.36	0.264	0.792
	One week after intervention	51.78±5.78	49.07±5.71	2.335	0.022*
CD4^+^ (%)	Before intervention	31.61±4.38	31.56±4.37	0.057	0.955
	One week after intervention	30.09±4.21	29.06±4.57	11.299	<0.001*
CD8^+^ (%)	Before intervention	22.87±3.28	22.79±3.31	0.121	0.905
	One week after intervention	21.91±3.31	20.38±3.21	2.323	0.022*
CD4^+^/CD8^+^ (%)	Before intervention	1.48±0.21	1.44±0.17	1.036	0.303
	One week after intervention	1.31±0.19	1.43±0.23	-2.816	0.006*

Note: *P < 0.05 indicates statistically significant difference. χ^2^ is the statistic value of the Chi-square test, and t is the statistic value of the T-test. Abbreviation: cluster of differentiation (CD).

### Comparison of postoperative complications

The study group exhibited a significantly lower rate of postoperative complications compared to the control group (P < 0.05), as presented in Table 5.

**Table 5:** Comparison of complications between early enteral nutrition group and control group.

Parameter	Study group (n = 49)	Control group (n = 49)	χ^2^	*P*
Infection	0	2 (4.08%)		
Electrolyte disorder	0	2 (4.08%)		
Pain	2 (4.08%)	3 (7.35%)		
Total incidence	1 (2.04%)	7 (14.29%)	4.899	0.027*

Note: *P < 0.05 indicates statistically significant difference. χ^2^ is the statistic value of the Chi-square test, and t is the statistic value of the T-test.

### Comparison of gastrointestinal complications

The incidence of gastrointestinal complications in the study group was notably lower than that in the control group (P < 0.05), as outlined in Table 6.

**Table 6:** Comparison of gastrointestinal complications between the two groups.

Parameter	Study group (n = 49)	Control group (n = 49)	χ^2^	*P*
Nausea	0	2 (4.08%)		
Vomit	0	1 (2.04%)		
Abdominal bloating	0	1 (2.04%)		
Gastric retention	1 (2.04%)	0		
Diarrhea	1 (2.04%)	3 (6.12%)		
Reflux gastrointestinal reaction	1 (2.04%)	2 (4.08%)		
Total incidence	3 (6.12%)	9 (18.37%)	3.885	0.049

## Discussion

In recent years, the rapid development of the social economy has brought significant changes to people’s lifestyles and dietary habits, which unfortunately has led to a rise in the incidence of gallbladder stone diseases. This increase has had a substantial impact on patients’ overall health and quality of life [16]. The etiology of gallstones is multifaceted, often attributed to factors such as an unhealthy diet, lack of exercise, liver disease, obesity, and more. While most patients exhibit no obvious symptoms, some may experience epigastric discomfort, biliary colic, and other related symptoms. Cholecystectomy is the primary treatment for gallstones, and with the application of laparoscopic technology, its effectiveness has significantly improved. However, due to the invasiveness of the procedure, patients may experience prolonged hypoxia postoperatively, which can lead to ischemia and hypoxia of the gastrointestinal mucosa, ultimately affecting gastrointestinal function and slowing recovery [17]. Additionally, fasting after cholecystectomy can result in severe nutritional deficiencies, making it essential to employ reasonable methods to supplement nutrition to meet the body’s metabolic and recovery needs [18].

Parenteral nutrition is commonly used as a postoperative nutritional supplement to ensure sufficient nutrient intake. However, this approach can result in incomplete nutrient delivery and complications that may hinder recovery [19]. In contrast, enteral nutrition support supplies necessary nutrients through the gastrointestinal tract, which aligns with the body’s physiological processes, and has shown promising therapeutic effects [20]. The findings of this study indicate that, compared to routine enteral nutrition support, early enteral nutrition support effectively improves the nutritional status, gastrointestinal function, and immune function of postoperative patients with cholecystolithiasis, while also reducing the incidence of treatment complications.

Total protein (TP), which consists of albumin and globulin, plays important physiological roles, including maintaining colloidal osmotic pressure, regulating blood pH, transporting metabolites, and enhancing immune and nutritional functions [21]. Prealbumin (PAB), also known as transthyretin (TTR), is synthesized by liver cells and is highly sensitive to detecting protein malnutrition and liver dysfunction due to its short half-life [21]. Albumin (ALB), the most abundant protein in plasma, is synthesized by liver parenchyma cells and is a key marker for evaluating nutritional status [21]. Transferrin (TF) is the primary iron-transporting protein in plasma, essential for the production of red blood cells [21]. The NRS 2002 and MUST scores are established tools used to assess the risk of malnutrition in hospitalized patients [22, 23]. Previous studies have demonstrated that early enteral nutrition can significantly enhance the nutritional status of postoperative cancer patients, reduce complications, and expedite recovery [24, 25]. In our study, the study group showed higher levels of TP, PAB, ALB, TF, and lower NRS 2002 and MUST scores compared to the control group, suggesting that early enteral nutrition significantly improves nutritional status in patients after gallstone surgery.

Early enteral nutrition support, initiated within 24 hours post-surgery, accelerates recovery by promoting small intestine function, enhancing the synthesis and metabolism of essential nutrients, and restoring gastrointestinal function [26]. The results of our study revealed that the study group experienced shorter times to first anal exhaust, bowel sound recovery, first defecation, first meal, and overall hospital stay compared to the control group (P < 0.05), indicating the effectiveness of early enteral nutrition in restoring gastrointestinal function after surgery. This improvement may be due to the stimulation of gastric movement, enhanced peristalsis, and increased secretion of gastric acid and gastrointestinal hormones [27, 28].

The immune response, particularly involving CD3+, CD4+, CD8+, and CD4+/CD8+ cells, plays a critical role in the body’s defense mechanisms [29]. Early enteral nutrition has been shown to enhance immune function, reduce the expression of inflammatory factors, and improve postoperative outcomes [29, 30]. In our study, the study group exhibited significantly higher CD3+, CD4+, CD8+, and CD4+/CD8+ levels compared to the control group (P < 0.05), affirming the beneficial impact of early enteral nutrition on immune function following gallstone surgery [31]. Early enteral nutrition also helps maintain gut microbiota stability and prevents bacterial translocation, further reducing the risk of complications [32].

Our findings showed that early enteral nutrition support was associated with a lower incidence of postoperative complications compared to the control group (P < 0.05), suggesting its efficacy in minimizing complications after gallstone surgery [33]. While some studies have raised concerns about early enteral nutrition increasing complications in certain fragile populations [34], our results support its application in carefully monitored patients, providing a theoretical basis for standardized nutritional support programs for postoperative patients with cholecystolithiasis.

Although this study highlights the advantages of early enteral nutrition in enhancing recovery, certain limitations exist. This retrospective study had a small sample size and did not group patients according to age, which may introduce selection bias. Future prospective studies with larger sample sizes are needed to confirm these findings.

## Conclusion

In summary, early enteral nutrition support can significantly improve the nutritional status of patients after gallstone surgery, including promoting gastrointestinal and immune function, and reducing the occurrence of complications, which has important clinical significance and value.

## Supporting information

S1 Checklist Human participants research checklist. (DOCX)
